# Estimation of Oncologic Surgery Case Volume Before and After the COVID-19 Pandemic in France

**DOI:** 10.1001/jamanetworkopen.2022.53204

**Published:** 2023-01-26

**Authors:** Christine Le Bihan-Benjamin, Mathieu Rocchi, Maxime Putton, Jean-Baptiste Méric, Philippe Jean Bousquet

**Affiliations:** 1 Health Data and Assessment Department, Survey Data Science and Assessment Division, National Cancer Institute, Boulogne-Billancourt, France; 2 Care Paths Organization Department, Public Health Division, National Cancer Institute, Boulogne-Billancourt, France; 3 Public Health Division, National Cancer Institute, Boulogne-Billancourt, France; 4 Survey Data Science and Assessment Division, National Cancer Institute, Boulogne-Billancourt, France; 5 Aix Marseille University, INSERM, IRD, Economics and Social Sciences Applied to Health & Analysis of Medical Information, Marseille, France

## Abstract

**Importance:**

COVID-19 has had a major effect on health care activities, especially surgery. At first, comparisons were proposed using 2019 activities as the highest standard. However, while such an approach might have been suitable during the first months of the pandemic, this might no longer be the case for a longer period.

**Objective:**

To examine approaches that may better assess the use of cancer surgeries.

**Design, Setting, and Participants:**

In a cross-sectional design, the nationwide French hospital facility data (Medicalised Information System Program) were used to assess cancer surgery for 6 cancer site categories in adults from January 1, 2010, to December 31, 2021.

**Exposure:**

Estimated cancer surgery activity during the COVID-19 pandemic.

**Main Outcomes and Measures:**

Three models were proposed to assess the expected number of surgical procedures between 2020 and 2021 and make a comparison with those observed in earlier years.

**Results:**

In France, cancer removal surgeries account for approximately 7000 hospitalizations per year for liver cancer; 4000 for pancreatic cancer; 7700 for ovarian cancer; 1300 for esophagus cancer; 23 000 for ear, nose, and throat (ENT) cancer; 78 000 for breast cancer; and 16 600 for thoracic cancers. For most cancer sites, the number of surgical procedures increased from 2010 to 2019: liver, 14%; pancreas, 38%; ovary, 14%; esophagus, 18%; breast, 8%; and thoracic, 29%. Assuming stability, these values underestimate the gap in activity observed in 2020-2021. For other procedures, a decrease was observed: stomach, −10%, and ENT, −6%. Assuming stability, these values overestimate the gap in activity observed in 2020-2021. At the end of 2021, according to the model, the gap in activity observed in 2020-2021 was estimated at between −1.4% and 1.7% for breast, −6.6% and −7.3% for thoracic, −3.1% and −2.5% for ovarian, −4.2% and −1.7% for pancreas, −6.7% and 5.9% for stomach, and −13.0% and −13.9% for esophageal cancers. For ENT, liver, and urologic cancers, because the trend was different before and after 2015, it was necessary to opt for modeling using only the most recent period. The cumulative gap in activity observed in 2020-2021 was estimated at −1.0% for ENT cancers, −5.3% for liver cancers, and −2.9% for urologic cancers.

**Conclusions and Relevance:**

The findings of this study suggest that short- and medium-term trends must be considered to estimate COVID-19 cancer surgery activities. Breast cancer is the site for which the activity showed the smallest decrease during the pandemic, with almost full recovery in 2021.

## Key Points

**Question:**

How can the association between COVID-19 and the number of cancer surgeries be analyzed?

**Findings:**

In this cross-sectional study using nationwide French hospital facility data (Medicalised Information System Program) from 2010 to 2021, 3 models to assess expected surgeries for cancer between 2020 and 2021 were proposed and compared with the numbers performed in earlier years. The models provided different estimates of expected activities compared with stable estimates, such as for 2019; breast cancer was the site with the smallest change during the pandemic, with almost full recovery in 2021.

**Meaning:**

The findings of this study suggest that trend analysis is especially important when disruption is observed and it may be best to consider short- and medium-term trends and prepare models on a site-by-site basis, because trends appear to differ among cancer sites.

## Teaser

This cross-sectional study examines models designed to estimate trends in the number of surgeries performed for treatment of cancer during the COVID-19 pandemic in France.

## Introduction

The COVID-19 pandemic that hit the world in 2020-2021 has had a major impact on the health care system in the countries affected. The pandemic has resulted in various degrees of public lockdown, travel bans, restrictions, and other limitations. For instance, France has experienced 3 periods of restrictions (or lockdown) and several additional waves of COVID-19 (eFigure in Supplement 1). The measures put in place by the government have evolved. The first lockdown (from March 17 to May 11, 2020) was very strict with a travel ban; the closure of schools, so-called nonessential retail and other businesses, and recreational facilities; and implementation of extensive remote working. The second (from October 28 to December 15, 2020) and third (from April 3 to May 2, 2021) lockdowns were less restrictive (schools stayed open and a limited number of businesses continued to operate). Between these lockdown periods, some French departments or the entire country were placed under curfew, depending on the period.

These measures had a marked influence on health care, resulting in cancellation or postponement of nonurgent care, such as surgery.^1^ In addition, during the first lockdown, invitations for screening programs (breast, colorectal, cervical, or lung cancer when proposed) were suspended,^2,3,4,5^ resulting in deferrals of treatments directly linked with cancer. These screening-related measures were not renewed during subsequent lockdown periods.

As a result, public health authorities developed programs to steer and propose solutions for people with cancer or those participating in screening.^6,7,8,9,10^ For instance, ministry of health and other national health agencies quickly set up systems to track care-related activity for people with cancer: from screening and diagnosis to treatment with surgery, drugs, or radiotherapy. As the highest standard, the number of procedures performed each month was compared with the number of procedures performed in the same month in 2019.^11,12,13^ This made it possible to quickly assess the association between the crisis and diagnosis and treatment of people with cancer in order to adapt the strategies developed at national and regional levels. Hence, a substantial decrease in cancer removal surgery activity was observed during the first wave.^11,14^ It was followed by subsequent effective safeguarding of these surgical procedures, thanks to the recommended actions, and incomplete restoration of activities, suggesting that patients not undergoing surgery in the first wave subsequently presented with more advanced tumors that were no longer suitable for surgery. However, while the latter assumption could be made for the first wave, it should not be true for subsequent waves, and normal activities should be observed.

Conversely, cancer incidence is evolving as a result of the combined effects of an aging population and changing risks, which naturally lead to a variation in the number of people treated each year.^15,16^ Hence, in many Western countries, some cancers have seen an increase over the past decade (eg, breast, liver, or pancreas), while others have decreased (eg, stomach or ovary).

These assumptions, combined with the length of the pandemic, call into question the highest standard (2019) considered for comparisons. Other estimates could be proposed to better assess expected activities. These estimators must be cost-effective for public health policy and need to meet certain criteria such as availability or ease of use.

Our objective was to revise the estimate of the differences between observed cancer surgery activity in 2020-2021 and expected activity by taking evolutionary activity trends in previous years into account.

## Methods

### Ethics

All methods were carried out in accordance with relevant guidelines and regulations. Data were deidentified before performing analyses. Access to the Medicalised Information System Program (PMSI) data is subject to authorization from CNIL (French data protection authority)—decree of December 26, 2016, No. 2016-1871. This study followed the Strengthening the Reporting of Observational Studies in Epidemiology (STROBE) reporting guideline. Data on the activity of all French hospitals are collected for billing purposes. They are also widely used for epidemiological studies. People are informed of this collection during their hospitalization. The data are made available to authorized users on a platform that the French National Cancer Institute is authorized to access.

### Data Source

This clinical setting data study is based on deidentified nationwide French hospitalization data collected by all French hospital facilities (PMSI) and processed on the secure data platform of the French Agency for Information on Hospital Care. For each hospital stay, information such as age, sex, date, diagnoses (coded in *International Statistical Classification of Diseases and Related Health Problems, 10th Revision*), and type of surgical procedure (coded in Classification Commune des Actes Medicaux) are provided. The French National Cancer Institute is authorized to access these data.

### Study Design and Population

Surgical removal activities among adults were evaluated for the 6 cancer site categories for which this activity is subject to authorization and minimum activity thresholds in France: digestive tract (stomach, liver, pancreas, and colorectal); gynecologic (ovarian); breast; chest; urologic; ear, nose, and throat (ENT); and maxillofacial cancers. Esophageal cancers are excluded from digestive tract, chest, and ENT cancers and presented separately for a better understanding.

### Measures

Stays with cancer removal surgery were identified by coding cancer as the main diagnosis (invasive or in situ) and a surgical removal procedure for people aged 18 years and older. Therefore, only histologically confirmed cancers were considered. Endoscopic ablations were not taken into account. The activities were assessed from January 1, 2010, to December 31, 2021.

### Statistical Analysis

Three scenarios to define the expected activities were proposed and compared. Scenario 1 comprised activity comparable to 2019: this is the scenario most commonly applied.^11,12,13^ Scenario 2 comprised modeling of expected activity in 2020-2021 based on the trend from 2010 to 2019. This approach accounts for changes in medium-term trends and extends them to the projections. Scenario 3 comprised modeling of expected activity in 2020-2021 based on data from 2015 to 2019. This approach builds on recent changes.

In scenarios 2 and 3, a linear regression model was fitted for each location assuming a normal distribution of the data: the number of hospitalizations is the dependent variable, and the year is the only independent variable. The projected activity and 95% CI in 2020-2021 were estimated according to these 2 models.

All statistical analyses were performed using WPS Analytics, version 4.0 (World Programming System). Figures were prepared using R, version 4.0.2 (R Foundation for Statistical Reporting).

## Results

Each year in France, 1.25 million people are hospitalized for cancer-related care (diagnosis, treatment, complications, or associated care), with a total of 7.5 million hospitalizations (including chemotherapy and radiotherapy sessions). Cancer removal surgeries account for approximately 78 000 hospitalizations for breast cancer, 57 000 for digestive cancers (including 36 600 for colorectal, 7000 for liver, 4000 for pancreas, 3200 for stomach, and 1300 for esophagus), 23 000 for ENT cancers, 16 600 for thoracic cancers, and 19 000 for gynecologic cancers (including 7700 for ovarian cancer).

### Activities in 2019, 2020, and 2021

Compared with the activity in 2019, cancer removal activity associated with the different sites evolved differently in 2020 and 2021. While, for all sites, there was a substantial decrease between March and May 2020, activity subsequently recovered to a greater or lesser degree depending on the site (eTable 1 in Supplement 1).

In 2020, cancer surgery activity decreased compared with 2019 for all sites, from −1.9% (ovary) to −11.8% (esophagus) (Table 1). In 2021, surgical activity was higher than in 2019 for several cancers (pancreas, 5.7%; breast, 5.0%), but for others it remained lower (stomach, −8.5%; esophagus, −6.5%).

**Table 1.** Number of Hospitalizations Observed in France From PMSI 2020-2021 Compared With 2019

Cancer	2019, No.	2020		2021	
		No.	Deviation from 2019, No. (%)	No.	Deviation from 2019, No. (%)
Breast	77 695	74 194	−3501 (−4.5)	81 610	3915 (5.0)
ENT	23 437	21 402	−2035 (−8.7)	22 329	−1108 (−4.7)
Thoracic	16 683	15 902	−781 (−4.7)	16 734	51 (0.3)
Ovary	7755	7608	−147 (−1.9)	7946	191 (2.5)
Liver	6977	6508	−469 (−6.7)	6853	−124 (−1.8)
Pancreas	3951	3810	−141 (−3.6)	4176	225 (5.7)
Stomach	3211	2916	−295 (−9.2)	2937	−274 (−8.5)
Esophagus	1343	1184	−159 (−11.8)	1256	−87 (−6.5)
Colorectal cancer	36 663	34 079	−2584 (−7.0)	35 718	−945 (−2.6)
Urologic cancer	41 410	39 930	−1480 (−3.6)	42 251	841 (2.0)

Abbreviations: ENT, ear, nose, and throat; PMSI, Medicalised Information System Program.

### Trend in Activities Between 2010 and 2019

**Figure 1.**
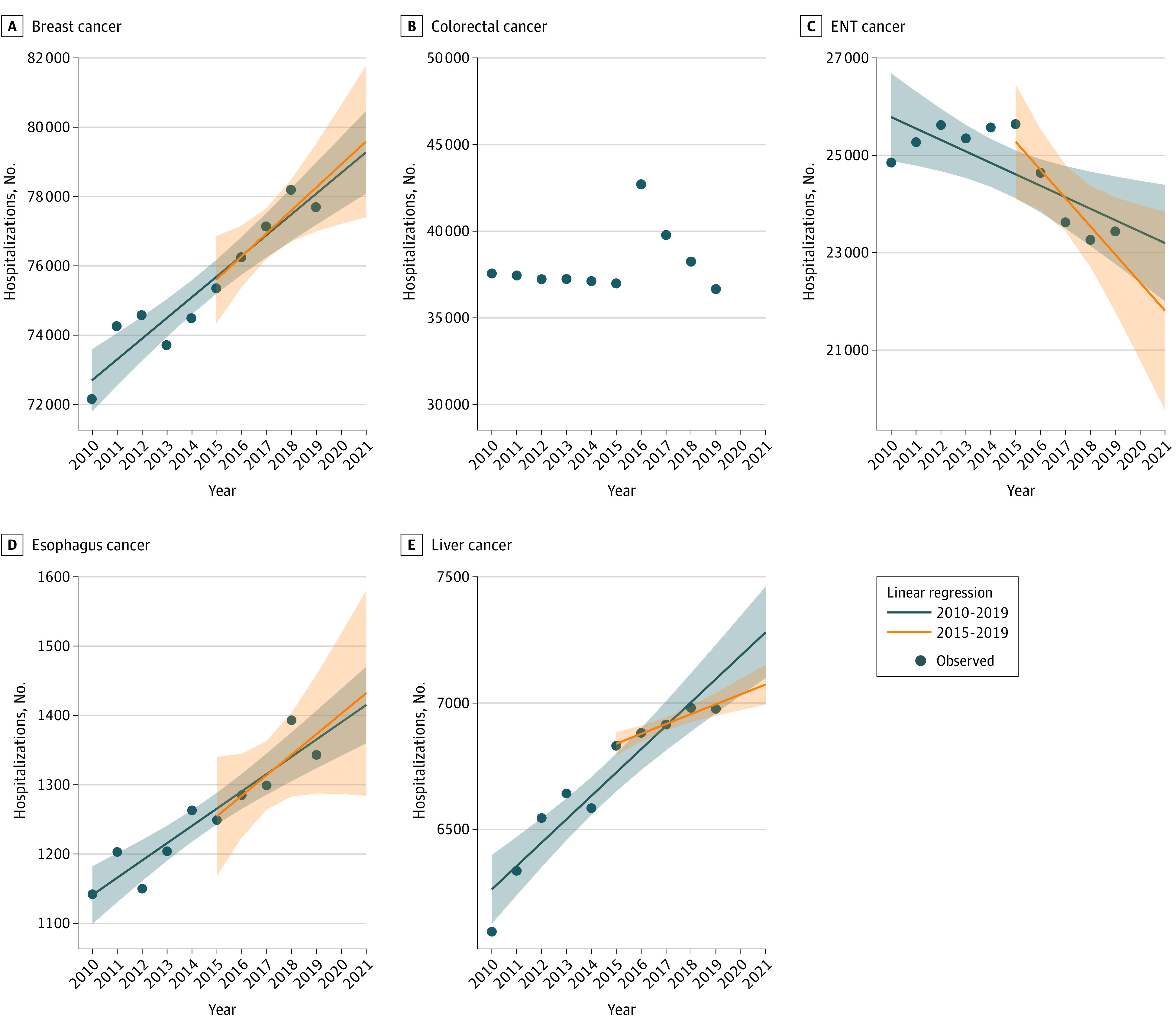
Modeling the Number of Cancer Surgeries in 2020-2021 According to 2010-2019 and 2015-2019 Periods for Breast; Ear, Nose, and Throat (ENT); Esophagus; and Liver Cancer Number of hospitalizations for surgeries performed for breast (A), colorectal (B), ENT (C), esophagus (D), and liver (E) cancer. Estimates for 2020 and 2021 based on the models; no modeling was performed for colorectal cancer. Shaded errors indicate 95% CIs.

**Figure 2.**
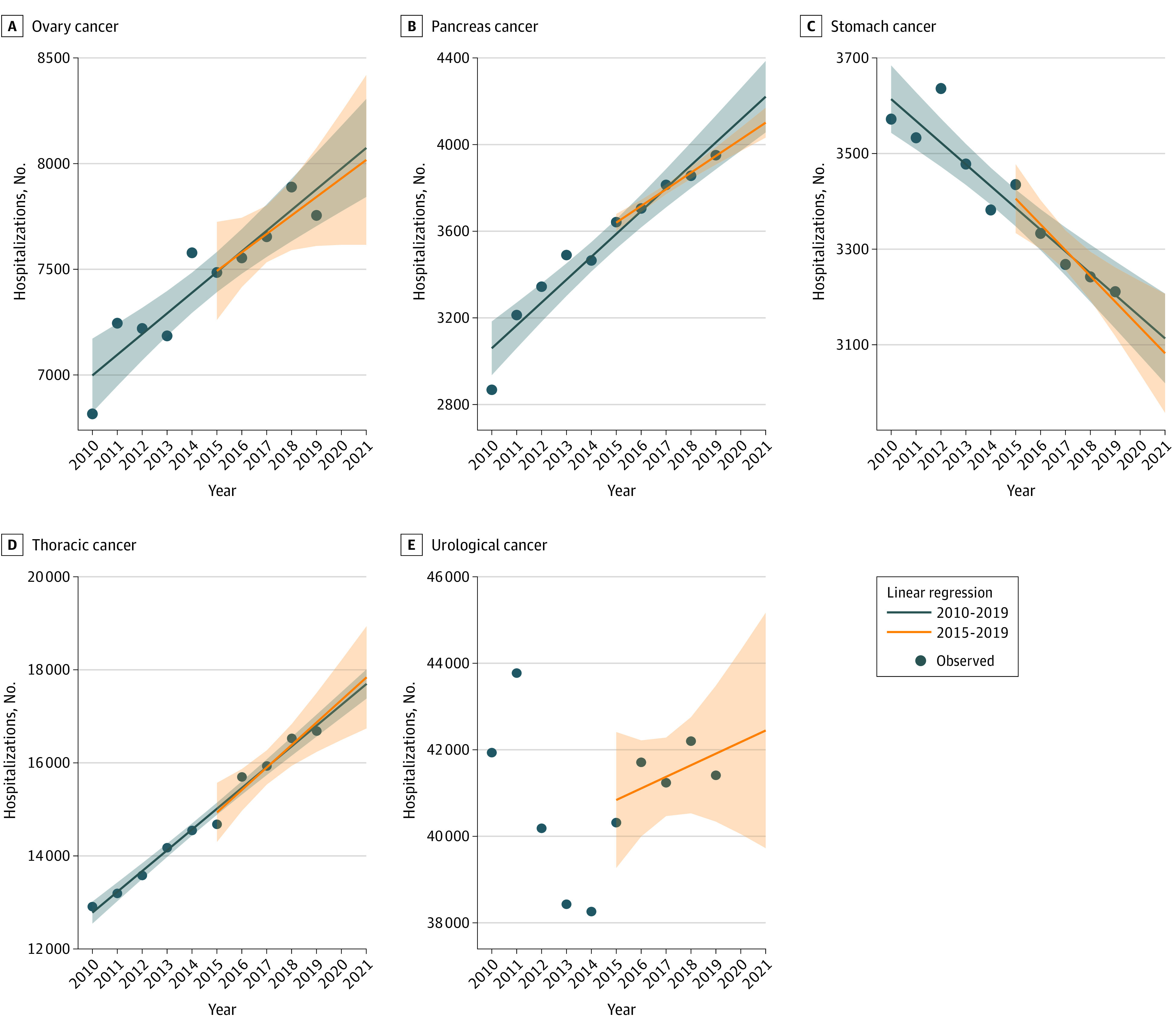
Modeling the Number of Cancer Surgeries in 2020-2021 According to 2010-2019 and 2015-2019 for Ovary, Pancreas, Stomach, Thoracic, and Urologic Cancer Number of hospitalizations for surgeries performed for ovary (A), pancreas (B), stomach (C), thoracic (D), and urologic (E) cancers. Estimates for 2020 and 2021 based on the models. Shaded errors indicate 95% CIs.

For most sites, the number of surgical procedures increased from 2010 to 2019 (Figure 1 and Figure 2; eTable 2 in Supplement 1): liver (14%), pancreas (38%), ovary (14%), esophagus (18%), breast (8%), and thoracic (29%) cancers. For others, a decrease was observed: stomach (−10%) and ENT (−6%). Some specific trends were observed. For colorectal cancers, the number was slightly lower in 2019 than in 2010 (−2%), but a sharp increase occurred in 2016. For urologic cancers, the number was slightly lower in 2019 than in 2010 (−1%), but a decrease in 2012-2013 was followed by a more recent increase.

Given the trends observed in previous years (Figure 1 and Figure 2), stability compared with 2019 did not appear to be a realistic assumption, except perhaps for liver cancer. It was assumed that a single model based on 2019 activities would not be the best or universal model. Hence, specific models were applied for each site and scenario.

### Models

When the number of surgeries increased between 2010 and 2019 (for all cancers except ENT and stomach), assuming stability underestimates the gap in activity observed in 2020-2021. Conversely, for ENT and stomach cancers, assuming stability overestimates the gap in activity observed in 2020-2021.

For most sites, the trend was homogeneous over the years (no change in slope), the estimates were very similar when using 2010-2019 (scenario 2) or 2015-2019 (scenario 3) to simulate 2020-2021 activity (Table 2), but appeared to be more accurate using 2010-2019, with the observed period being longer. Therefore, there is little difference in the cumulative gap observed in 2020-2021 between the 2 projection models.

**Table 2.** Observed Number of Hospitalizations in France From PMSI in 2019-2021 and Expected Number of Hospitalizations in 2020-2021 According to Scenarios 1, 2, and 3^a^

Cancer type	O, No.	Scenario		
		1, No. (%)	2, No. (95% CI)	3, No. (95% CI)
**Breast**				
2019	77 695	NA	NA	NA
2020	74 194	77 695	78 671 (76 827 to 80 515)	78 914 (76 559 to 81 271)
2021	81 610	77 695	79 269 (77 335 to 81 203)	79 577 (76 857 to 82 298)
Change, No. (%)				
O to E 2020	NA	−3501 (−4.5)	−4477 (−5.7)	−4720 (−6.0)
O to E 2021	NA	3915 (5.0)	2341 (3.0)	2033 (2.6)
O to E 2020 + 2021	NA	414 (0.3)	−2136 (−1.4)	−2687 (−1.7)
**ENT**				
2019	23 437	NA	NA	NA
2020	21 402	23 437	23 433 (21 584 to 25 282)	22 386 (20 184 to 24 588)
2021	22 329	23 437	23 197 (21 259 to 25 136)	21 808 (19 265 to 24 350)
Change, No. (%)				
O to E 2020	NA	−2035 (−8.7)	−2031 (−8.7)	−984 (−4.4)
O to E 2021	NA	−1108 (−4.7)	−868 (−3.7)	521 (2.4)
O to E 2020 + 2021	NA	−3143 (−6.7)	−2899 (−6.2)	−463 (−1.0)
**Thoracic**				
2019	16 683	NA	NA	NA
2020	15 902	16 683	17 250 (16 764 to 17 736)	17 354 (16 169 to 18 540)
2021	16 734	16 683	17 697 (17 188 to 18 207)	17 838 (16 469 to 19 207)
Change, No. (%)				
O to E 2020	NA	−781 (−4.7)	−1348 (−7.8)	−1452 (−8.4)
O to E 2021	NA	51 (0.3)	−963 (−5.4)	−1104 (−6.2)
O to E 2020 + 2021	NA	−730 (−2.2)	−2311 (−6.6)	−2556 (−7.3)
**Ovary**				
2019	7755	NA	NA	NA
2020	7608	7755	7976 (7618 to 8335)	7930 (7496 to 8364)
2021	7946	7755	8074 (7698 to 8450)	8017 (7517 to 8518)
Change, No. (%)				
O to E 2020	NA	−147 (−1.9)	−368 (−4.6)	−322 (−4.1)
O to E 2021	NA	191 (2.5)	−128 (−1.6)	−71 (−0.9)
O to E 2020 + 2021	NA	44 (0.3)	−496 (−3.1)	−393 (−2.5)
**Liver**				
2019	6977	NA	NA	NA
2020	6508	6977	7188 (6908 to 7468)	7035 (6949 to 7120)
2021	6853	6977	7280 (6987 to 7574)	7074 (6975 to 7172)
Change, No. (%)				
O to E 2020	NA	−469 (−6.7)	−680 (−9.5)	−527 (−7.5)
O to E 2021	NA	−124 (−1.8)	−427 (−5.9)	−221 (−3.1)
O to E 2020 + 2021	NA	−593 (−4.2)	−1107 (−7.7)	−748 (−5.3)
**Pancreas**				
2019	3951	NA	NA	NA
2020	3810	3951	4115 (3860 to 4370)	4024 (3950 to 4099)
2021	4176	3951	4221 (3954 to 4488)	4101 (4015 to 4187)
Change, No. (%)				
O to E 2020	NA	−141 (−3.6)	−305 (−7.4)	−214 (−5.3)
O to E 2021	NA	225 (5.7)	−45 (−1.1)	75 (1.8)
O to E 2020 + 2021	NA	84 (1.1)	−350 (−4.2)	−139 (−1.7)
**Stomach**				
2019	3211	NA	NA	NA
2020	2916	3211	3159 (3014 to 3304)	3136 (3002 to 3270)
2021	2937	3211	3113 (2961 to 3265)	3082 (2927 to 3237)
Change, No. (%)				
O to E 2020	NA	−295 (−9.2)	−243 (−7.7)	−220 (−7.0)
O to E 2021	NA	−274 (−8.5)	−176 (−5.7)	−145 (−4.7)
O to E 2020 + 2021	NA	−569 (−8.9)	−419 (−6.7)	−365 (−5.9)
**Esophagus**				
2019	1343	NA	NA	NA
2020	1184	1343	1390 (1305 to 1476)	1403 (1243 to 1563)
2021, No. (95% CI)	1256	1343	1415 (1325 to 1505)	1432 (1247 to 1617)
Change, No. (%)				
O to E 2020	NA	−159 (−11.8)	−206 (−14.8)	−219 (−15.6)
O to E 2021	NA	−87 (−6.5)	−159 (−11.2)	−176 (−12.3)
O to E 2020 + 2021	NA	−246 (−9.2)	−365 (−13.0)	−395 (−13.9)
**Colorectal**				
2019	36 663		NA	NA
2020	34 079	36 663	NA	NA
2021	35 718	36 663	NA	NA
Change, No. (%)				
O to E 2020	NA	−2584 (−7.0)	NA	NA
O to E 2021	NA	−945 (−2.6)	NA	NA
O to E 2020 + 2021	NA	−3529 (−4.8)	NA	NA
**Urologic**				
2019	41 410	NA	NA	NA
2020	39 930	41 410	NA	42 178 (39 241 to 45 116)
2021	42 251	41 410	NA	42 446 (39 054 to 45 838)
Change, No. (%)				
O to E 2020	NA	−1480 (−3.6)	NA	−2248 (−5.3)
O to E 2021	NA	841 (2.0)	NA	−195 (−0.5)
O to E 2020 + 2021	NA	−639 (−0.8)	NA	−2443 (−2.9)

Abbreviations: E, expected; NA, not applicable; O, observed; PMSI, Medicalised Information System Program.

^a^ Scenario 1 comprised activity comparable to 2019, scenario 2 comprised modeling of activity in 2020-2021 based on the trend from 2010 to 2019, and scenario 3 comprised modeling of activity in 2020-2021 based on the trend from 2015 to 2019.

At the end of 2021, the gap in activity observed in 2020-2021 was estimated at between −1.4% and 1.7% for breast, −6.6% and −7.3% for thoracic, −3.1% and −2.5% for ovarian, −4.2% and −1.7% for pancreas, −6.7% and 5.9% for stomach, and −13.0%, and −13.9% for esophageal cancers.

Some specificities were also observed. Due to an atypical trend, no modeling other than scenario 1 was applied for colorectal cancers. For ENT, liver, and urologic cancers, change in slopes were observed in 2015. For ENT cancers, activity was almost stable between 2010 and 2015, then decreased from 2016. For liver cancer, growth slowed from 2015 onward. However, since 2015, the trend has been regular. For these 2 sites, it was necessary to opt for modeling using only the most recent period. For urologic cancers, before 2015, the trend was too irregular to be taken into account in a model; thus, scenario 2 was not applied and it was necessary to opt for modeling using only the most recent period. At the end of 2021, the cumulative gap in activity observed in 2020-2021 was estimated at −1.0% for ENT cancers, −5.3% for liver cancers, and −2.9% for urologic cancers.

## Discussion

Due to demographic trends, changes in diagnostic and treatment procedures, and other cancer surgery-related activities were not stable over periods of several years. It is therefore necessary to consider trends in activity over the past several years. The trends provide different estimates of expected activities compared with stable estimates, such as for 2019. The consideration of the trend is to be handled with care in case of disruption.

### Modeling

Hence, for a better understanding, activity trends must be modeled. Several methods are used to model and predict cancer incidence over time. Some of these, as reported in the article by Møller et al,^17^ are based on the age-period-cohort model (Poisson regression), while others are based on linear, nonlinear, or smoothed versions of age-period-cohort models. More recently, Uhry et al^18^ proposed a multidimensional penalized spline model. Globocan^16^ reports trends over time in 185 countries using, where available, the Dyba and Hakulinen Poisson regression method.^19^ The Surveillance, Epidemiology, and End Results Program uses joinpoint regression.^20^

There is no universal method for choosing which model to use to estimate the expected activity. Unlike these complex models used to project cancer incidence or surgery shortfalls,^21^ the models proposed in the present study are very simple (based only on temporal changes) and easy to use. They are based on linear regression (assuming a normal distribution). We propose a pragmatic choice based on data observation. If there is no break in the evolution, taking a 5- or 10-year step back leads to very close estimates, but more precise if the period is longer. However, in the case of a nonhomogeneous evolution, the estimation is more delicate and will depend on the explicability and the reason for the evolution. In some situations, such as colorectal cancer, it may be preferable to not carry out modeling.

### Results of the Models

Applying these considerations to the pandemic period, the surgical activity observed in 2020 appeared to be substantially lower than that expected, regardless of the modeling applied, except for ovarian, ENT, and urologic cancers with scenario 3 (modeling based on data from 2015 to 2019). For 2021, surgical activity was within estimations for ENT, ovarian, and pancreatic cancer, suggesting a return to normal activity without recovery. Breast cancer was the only site with substantially higher activity than expected in 2021 (scenarios 2 and 3), resulting in the smallest cumulative shortfall over 2020-2021. In addition, the estimated activity of breast cancer was below that for thoracic, liver, stomach, and esophageal cancers, inflating the influence of the COVID-19 pandemic. For breast, ENT, and pancreas cancers, the cumulative gap in 2020-2021 was less than the cumulative shortfall in 2020, which suggests a partial recovery.

From an epidemiological point of view, changes in activity between 2010 and 2019 are consistent with incidence trends for most cancers (breast, chest, pancreas, ENT, stomach, and urologic). Some specificities must also be considered. For instance, the variation of prostate cancer incidence in France, as in many other countries, is mainly associated with changes in individual screening practices commencing with prostate-specific antigen measurement followed by a biopsy.^22^ A major increase was followed by a rapid decline in incidence between 2005 and 2020. Nowadays, a slight increase is observed, similar to that observed prior to the change. For colorectal cancer, a decrease in incidence was observed in 2016-2017, mainly in individuals aged 50 to 70 years. This trend reflected the change in screening test (fecal immunochemical test) that took place in 2016.

From a clinical point of view, as observed in many countries,^8,23^ the discontinuation of screening programs and the reorganization of surgical services to focus on critical care caused large reductions in surgical treatments. The activity differentials observed are probably linked to COVID-19. However, it is unknown why there are different scenarios in terms of the cumulative gap at the end of 2021 for different cancer types. It is unlikely that any innovations during this period resulting in changes in treatment regimens occurred (eg, intervals observed between 2010 and 2019 for some surgeries). The unavailability of anesthetists, anesthetic products, and hospital beds may nevertheless have led to changes in practice,^24^ and most professional societies issued guidelines for management tailored to the pandemic period.^25,26,27,28,29,30,31,32^

Breast cancer surgery showed the smallest shortfall, with almost full recovery in 2021, even when taking into account the increasing trend in recent years. This type of cancer has a good prognosis, is accessible to screening, and surgical removal is systematic except for metastatic stages; there is also benefit from outpatient surgery, which uses fewer inpatient beds. The rate of outpatient surgery for breast-conserving surgery has been increasing for several years,^33^ and continued to increase during this pandemic period (51.7% in 2019, 56.0% in 2020, and 58.5% in 2021). In the Netherlands, where breast cancer incidence declined between 2018-2019 and 2020, except for stage IV, treatments differed depending on the periods in 2020,^34^ with more patients receiving primary hormonal treatment as recommended.^25,26^

For other sites, therapeutic alternatives put in place over these 2 years may have led to surgery not being performed. Surgery for esophageal cancer requires many resources and often requires admission to critical care. Thus, alternative treatment options, such as definitive chemoradiotherapy, might have been recommended preferentially.^32^ Hence, to better understand the impact of the pandemic on cancer treatment, the entire care trajectory (combination of surgery, drug treatment, and radiotherapy) must be explored.

Some authors raise the issue of recovery time.^21,35^ This assumes that people without surgical treatment can still undergo operations at a later time. In addition to the total number of tumor removals, the issue of the timeframe for carrying out surgery and the type of surgery performed may have changed over the months, which is not presented in this analysis.

These results concern the whole of France. There may be age-related or regional disparities with some groups being more affected than others. If necessary, modeling could be carried out for specific age groups or at a regional level. Some cohorts may have been differentially impacted by COVID-19.

### Limitations

This study has limitations. The models do not consider age, sex, or incidence trends, but nonetheless, the estimated activities appear to be more realistic in terms of change patterns. For the purposes of steering the organization of care annually, in view of public health objectives and given the major impact of COVID-19 on health care, it does not seem appropriate to develop a sophisticated model for estimating expected activity. A rapidly implemented method is more useful because it is essential from a public health point of view that decision-makers can have an estimate of the loss of care in the case of an exceptional situation at the scale of a country or a region.

For some sites, such as the liver, a more complex polynomial model could have been used, but given the data, the estimates would have been very similar to scenario 1. Because this was a retrospective use of the data to estimate the volume of unperformed activity, the activity was analyzed annually. For a prospective use of surveillance, time series modeling^3,21,36^ would be more appropriate.

## Conclusions

The findings of this study suggest that short- and medium-term trends must be considered and modeling needs to be done on a site-by-site basis to gain a better understanding of health care activities for treatment of cancer. Reporting based on 2019 activities leads to misunderstandings and can give rise to inappropriate health policy decisions. As expected, further work on care trajectories for cancer treatment (combination of surgery, drug treatment, and radiotherapy) is needed, as well as long-term observations of people treated during this period, to fully investigate the influence on survival, adverse effects, and recurrence.

## Notes

Supplement 1. **eFigure.** COVID-19 Epidemic in France (2020-2021) **eTable 1.** Number of Hospitalizations Observed by Month and Year **eTable 2.** Number of Hospitalizations Observed by Year From 2010 to 2021

Supplement 2.
Data Sharing Statement
