# Supplementary material for: Estimation of Oncologic Surgery Case Volume Before and After the COVID-19 Pandemic in France
Source: JAMA Netw Open. 2023 Jan 26;6(1):e2253204. doi: 10.1001/jamanetworkopen.2022.53204 (PMC9880797; doi:10.1001/jamanetworkopen.2022.53204)
Supplement: Supplement 1. — eFigure. COVID-19 Epidemic in France (2020-2021) eTable 1. Number of Hospitalizations Observed by Month and Year eTable 2. Number of Hospitalizations Observed by Year From 2010 to 2021 [file jamanetwopen-e2253204-s001.pdf]

Supplementary Online Content

Le Bihan-Benjamin C, Rocchi M, Putton M, Méric JB, Bousquet PJ. Estimation of oncologic surgery case volume before and after the COVID-19 pandemic in France. *JAMA Netw Open*. 2023;6(1):e2253204. doi:10.1001/jamanetworkopen.2022.53204

eFigure. COVID-19 Epidemic in France (2020-2021)

eTable 1. Number of Hospitalizations Observed by Month and Year

eTable 2. Number of Hospitalizations Observed by Year From 2010 to 2021

This supplementary material has been provided by the authors to give readers additional information about their work.

© 2023 Le Bihan-Benjamin C et al. *JAMA Network Open.*

eFigure: Covid-19 epidemic in France (2020-2021)

Based on the number of positive tests and hospitalisation rates, 5 successive waves can be identified between March 2020, and April 2022. The ratios between hospitalisations and number of positive tests were slightly lower for more recent waves. This was even more significant for the most recent wave (mainly due to the omicron variant).

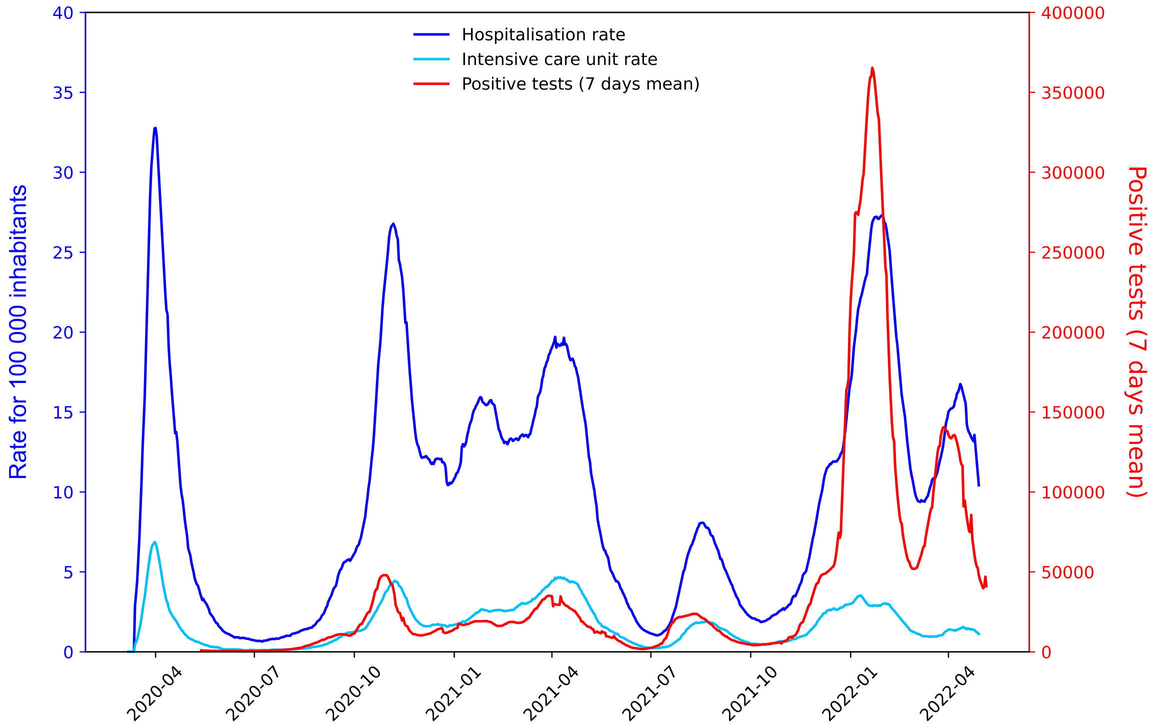

© 2023 Le Bihan-Benjamin C et al. *JAMA Network Open.*

eTable1: Number of hospitalizations observed by month and year

		Month											
**Cancer**	**Year**	**01**	**02**	**03**	**04**	**05**	**06**	**07**	**08**	**09**	**10**	**11**	**12**
Breast	2019	7036	6178	7025	6314	6845	6551	7103	5261	6270	6645	6309	6162
	2020	7203	6278	7150	5513	4241	5023	6343	5144	6712	6637	6977	6973
	2021	7251	6671	7609	7130	6687	7570	6913	5316	6801	6630	6229	6803
ENT	2019	1968	1836	2109	1956	2069	1896	2062	1545	1908	2071	2002	2026
	2020	2040	1915	1938	1402	1538	1683	1813	1469	1913	1916	1872	1903
	2021	1898	1873	2078	1935	1861	2040	1882	1393	1861	1942	1763	1803
Thoracic	2019	1290	1332	1554	1448	1562	1385	1536	1029	1426	1531	1320	1303
	2020	1355	1396	1465	1033	1278	1310	1270	1048	1380	1490	1406	1471
	2021	1356	1433	1557	1363	1439	1576	1356	1107	1367	1487	1339	1354
Ovary	2019	609	631	727	626	672	635	714	484	665	723	626	662
	2020	593	642	691	492	549	686	708	576	668	675	644	684
	2021	607	684	720	700	653	742	691	507	637	702	619	684
Liver	2019	544	595	654	618	585	618	605	422	576	667	545	564
	2020	517	576	628	450	478	583	577	424	523	596	546	610
	2021	519	574	654	622	606	640	612	382	519	625	547	553
Pancreas	2019	280	337	364	381	323	305	371	294	275	347	300	397
	2020	308	329	355	248	256	333	389	258	300	362	301	371
	2021	269	381	395	360	360	374	369	278	310	382	341	357

© 2023 Le Bihan-Benjamin C et al. *JAMA Network Open.*

Stomach	2019	251	254	297	270	262	266	296	216	248	312	270	270
	2020	244	271	276	193	217	266	278	173	203	260	252	283
	2021	236	230	266	241	241	271	243	198	241	267	245	258
Esophagus	2019	76	101	111	127	119	118	136	97	91	127	102	145
	2020	96	96	106	71	78	114	132	79	90	111	100	111
	2021	100	105	124	99	94	123	104	96	88	107	110	106
Colorectal cancer	2019	2770	3004	3257	3322	3279	2996	3561	2651	2718	3217	2824	3105
	2020	2614	2916	3426	2368	2326	2675	3125	2595	2788	3043	2939	3264
	2021	2693	2861	3288	3057	2970	3285	3358	2779	2814	3033	2631	2949
Urological cancer	2019	3411	3475	3902	3527	3692	3539	3575	2217	3575	3894	3259	3348
	2020	3594	3575	3546	2645	3408	3520	3148	2088	3449	3646	3689	3622
	2021	3612	3704	4000	3650	3587	3957	3498	2283	3713	3908	3084	3255

© 2023 Le Bihan-Benjamin C et al. *JAMA Network Open.*

eTable2: Number of hospitalizations observed by year from 2010 to 2021

	**2010**	**2011**	**2012**	**2013**	**2014**	**2015**	**2016**	**2017**	**2018**	**2019**	**2020**	**2021**
Breast	72 158	74 260	74 578	73 713	74 490	75 353	76 249	77 139	78 194	77 695	74 194	81 610
ENT	24 851	25 270	25 621	25 348	25 571	25 640	24 641	23 623	23 264	23 437	21 402	22 329
Thoracic	12 910	13 194	13 579	14 176	14 547	14 679	15 697	15 932	16 526	16 683	15 902	16 734
Ovary	6 816	7 245	7 220	7 185	7 578	7 485	7 554	7 654	7 889	7 755	7 608	7 946
Liver	6 095	6 336	6 545	6 642	6 584	6 831	6 882	6 915	6 981	6 977	6 508	6 853
Pancreas	2 868	3 213	3 344	3 490	3 465	3 642	3 705	3 814	3 856	3 951	3 810	4 176
Stomach	3 572	3 533	3 636	3 478	3 382	3 435	3 333	3 268	3 242	3 211	2 916	2 937
Esophagus	1 142	1 203	1 150	1 204	1 263	1 249	1 285	1 299	1 393	1 343	1 184	1 256
Colorectal cancer	37 562	37 445	37 229	37 240	37 121	36 987	42 707	39 779	38 248	36 663	34 079	35 718
Urological cancer	41 934	43 773	40 185	38 429	38 260	40 316	41 710	41 238	42 200	41 410	39 930	42 251

© 2023 Le Bihan-Benjamin C et al. *JAMA Network Open.*
