# Supplementary material for: Estimation of Oncologic Surgery Case Volume Before and After the COVID-19 Pandemic in France
Source: JAMA Netw Open. 2023 Jan 26;6(1):e2253204. doi: 10.1001/jamanetworkopen.2022.53204 (PMC9880797; doi:10.1001/jamanetworkopen.2022.53204)
Supplement: Supplement 2. — Data Sharing Statement [file jamanetwopen-e2253204-s002.pdf]

Data Sharing Statement

Le Bihan-Benjamin. Estimation of Oncologic Surgery Case Volume Before and After the COVID-19 Pandemic in France. *JAMA Netw Open*. Published January 26, 2023. doi:10.1001/jamanetworkopen.2022.53204

Data
Data available: No
